# Trends in Working Life Expectancy and Untapped Employment Potential in an Ageing Population: The Case of Germany

**DOI:** 10.1007/s10680-026-09773-x

**Published:** 2026-04-25

**Authors:** Harun Sulak, Christian Dudel, Elke Loichinger, Sebastian Klüsener

**Affiliations:** 1 Federal Institute for Population Research (BiB), Wiesbaden, Germany; 2 Max Planck Institute for Demographic Research, Rostock, Germany; 3 Max Planck – University of Helsinki Center for Social Inequalities in Population Health (MaxHel Center), Helsinki, Finland; 4 Federal Statistical Office of Germany (Destatis), Wiesbaden, Germany; 5 Department of Sociology and Social Psychology, University of Cologne, Cologne, Germany; 6 Vytautas Magnus University, Kaunas, Lithuania

**Keywords:** Working life expectancy, Socio-economic differences, Underemployment, Overemployment, Lifetime in unemployment, Germany

## Abstract

The population of Germany is ageing rapidly. As a result, the country’s working age population is expected to shrink substantially over the next 15 years. To cope with this challenge, a number of policy reforms have been introduced to activate untapped employment potential and increase the length of working life. However, little is known to what extent employment potentials have already been realised, and what differences exist between socio-economic groups. This paper addresses these questions using large-scale data from the German Microcensus to calculate working life expectancy (WLE) and unrealised working life expectancy. WLE not only accounts for labour market status (i.e., employed, unemployed, and inactive), but also considers actual working hours and preferences on working hours that allow capturing under- and overemployment. Our results show that WLE for most groups exhibits a U-shaped development, with WLE in 2022 resembling the levels recorded in 1991. At the same time, socio-economic inequalities in WLE were and still are substantial, and the group with the highest WLE works more than twice as long over their lifetime compared with the group with the lowest WLE. Moreover, trends in unrealised employment potential mirror trends in WLE – when WLE goes down, unrealised potential goes up. During recent years, only individuals with a low level of education have continued to display considerable unrealised potential, whereas for highly educated individuals, virtually no unrealised potential remains. On the contrary, we obtained evidence of overemployment for this group.

**Supplementary Information:**

The online version contains supplementary material available at 10.1007/s10680-026-09773-x.

##  Introduction

Population ageing poses challenges to labour markets and social security systems around the globe, with Germany being particularly affected. By 2035, the size of the German working-age population is expected to have declined by at least two million to around 48 million individuals (Federal Statistical Office of Germany, 2022). A major driver behind these trends is that the relatively large baby boom cohorts born in the 1950s and 1960s have begun to reach retirement age and leave the labour market, while the younger cohorts entering the labour market are considerably smaller. The shrinking labour force is already leading to labour supply shortages and this looks set to intensify in the near future, with adverse impacts on economic growth and declining contributions to the German social security system (e.g. Federal Institute for Population Research, 2019; Deutsche Bundesbank 2019). At the same time, a growing number of older individuals leads to increasing expenditure on pension benefits and the healthcare system.

One strategy to mitigate the consequences of population ageing is to identify and activate untapped employment potential in the population (OECD, 2023). However, there is a lack of coherent in-depth accounts that enable an assessment of this potential over time, both for the entire population as well as specific subgroups in Germany. The existing literature focuses primarily on individual phases of life such as the family phase or retirement age (Hess, 2018; Börsch-Supan et al., 2023), or on employment, unemployment and underemployment rates for broad age groups (Bell & Blanchflower, 2013; Duell & Vetter, 2020). However, only by looking at working life in its entirety is it possible to shed light on the employment of the population and individual population groups and to quantify age-specific and group-specific aspects of employment and employment potential that can be activated. Moreover, previously published calculations usually lack differentiated analyses based on socio-demographic characteristics or are instead based on headcounts and therefore do not account for differences in the hours worked.

In this paper, we build on high-quality data from the German Microcensus for the years 1991 to 2022 and employ the concept of working life expectancy (WLE) to quantify trends in employment and employment potential. WLE is the average lifetime spent in employment, and we extend this concept to also cover unrealised WLE. Unrealised WLE is the average lifetime which individuals would like to spend in employment, but fail to do so due to unemployment or underemployment. The sum of WLE and unrealised WLE is called potential WLE; i.e. the average lifetime spent in employment if there was no unemployment or underemployment. We estimate realised, unrealised and potential WLE at age 15 using modified period life tables, also taking into account working hours and preferences on working hours. For these life table calculations, we used employment information between the age of 15 and 74 years to create synthetic full working life trajectories. Results on unrealised and potential WLE can be calculated in full from 2008 onwards. Our outcomes are stratified by gender, region (eastern and western Germany), and educational attainment. In addition, we apply Kitagawa’s (Kitagawa, 1955) decomposition method to disentangle the factors underlying the differences in realised WLE and potential WLE between socio-economic groups. Finally, we break down WLE and potential WLE by age group, showing whether gains or losses in each indicator are occuring during specific life phases or age ranges, or across all age groups.

Germany is an interesting case for several reasons. First, the country’s population is ageing rapidly, and several policy measures have been introduced with the aim of extending working life and activating untapped employment potential. These policy measures include, for instance, increases in the statutory retirement age. Similar measures have been introduced in many other countries and our results might provide some indications regarding their potential impact. Second, the German labour market is highly stratified. For instance, in terms of examining untapped employment potential, the distinction between eastern Germany and western Germany is highly relevant. Untapped employment potential is particularly concentrated among women, and eastern and western Germany have a long history of differences in female employment (Becker et al., 2020); Lippmann et al., 2020). In addition, eastern and western Germany differed substantially in employment trends following reunification in 1990, which continues to affect current labour market participation to this day in certain cases. Studying this and other dimensions of inequality will show how big group differences can become in a highly stratified setting. Third, while the German Microcensus on which our results are based is a survey, it is in many respects different to other typical surveys. In particular, each wave covers around 1 per cent of all German households, thereby making the data set several magnitudes larger than typical surveys. Moreover, participation in the Microcensus is required by law and is compulsory, which implies that participation rates are high (usually far above 90 per cent). This makes the Microcensus a unique large-scale, high-quality data set.

This study contributes to the existing literature in two major ways, namely conceptually and empirically. First, WLE has to date only been studied in terms of realised employment. We adapt this concept to cover employment potential and argue that from a policy perspective, both realised WLE and potential WLE are crucial measures. The former informs policymakers of the status quo, while the latter shows, in an easy-to-understand way, what could be achieved and what socio-economic groups to target. Second, we extend existing empirical findings on WLE in Germany. While Germany has been the subject of some interest in recent literature (Beller et al., 2024; Dudel et al., 2023; Tetzlaff et al., 2022), research has largely focused only on very recent years or on specific age groups such as late working life, or employed smaller samples of the population. Here, we cover more than 30 years starting from 1991 and covering the entire age range of the working life, as well as introducing the new concept of unrealised and potential WLE and presenting complete results starting from 2008.

## Background

### Institutional Context and Employment Trends by Age

The institutional context plays a key role in shaping working trajectories and WLE (DiPrete, 2002). This concerns the age at which employment is taken up, employment during training phases, interruptions and restrictions to employment in the course of one’s working life, retirement age and possible employment beyond retirement age. All of this is based on the conditions of the labour market and the legal framework, as well as on social norms and values. Ultimately, these factors are often mutually dependent. In Germany, all of these elements have been subject to major changes over the last 30 years (Geyer et al., 2012; Wingerter, 2011).

Entry into the labour market is primarily influenced by the number of years spent in school and vocational training as well as by the labour market situation for career starters. In Germany, the proportion of people completing academic education has increased significantly over time (Federal Statistical Office of Germany, 2024c). Consequently, even though the abolition of conscription for men in 2011 and reforms in university education in general have contributed to the fact that university graduates are now younger than they were in the late 1990s and early 2000s, the significant decline in youth employment over time has seen the average age at which people enter the workforce rise sharply over the last 30 years (Elsholz et al., 2018; Federal Statistical Office of Germany, 2019, 2025).

Interruptions and restrictions in employment over the life course are often due to unemployment, or family obligations such as childcare and caring for close relatives. Germany has experienced two phases of particularly high unemployment in the last 30 years: in both the mid-1990s and the mid-2000s, unemployment stood at well above 10 per cent, peaking at 13 per cent in 2005 (Federal Statistical Office of Germany, 2024d).

Care for children and family members remains predominantly undertaken by women. Major reforms impacting employment interruptions due to family commitments were carried out primarily in the 2000s and 2010s. The introduction of a more substantial parental allowance in 2007, which replaced the previous child-raising allowance, led to mothers returning to the labour market earlier and had an overall positive effect on their labour market participation (e.g., Tamm, 2018; Bergemann & Riphahn, 2023; Geyer et al., 2012). The expansion of childcare facilities and the associated increase in the childcare rate for children under the age of 3 was an essential component of a higher labour force participation among mothers (Diener & Berngruber, 2018). In addition to the legal framework, other factors play a role in terms of employment interruptions and restrictions in Germany. These include social norms and role models in relation to the gender-specific division of labour in families in general and attitudes towards the organisation of childcare (Schulz & Blossfeld, 2006). Reductions in employment during childbearing ages also have a strong influence on career trajectories, meaning that labour market participation is also limited in later working life (Grunow et al., 2011). As a result, there is a delay until improvements in policies aimed at reconciling family and working life more effectively have an impact on the employment rates of mothers with older children. Furthermore, given these circumstances, it is particularly important to consider employment the entire age range of the working life.

The employment of older persons in Germany is closely linked to the statutory retirement age. Only a small proportion of employees remain in the labour market beyond this age. This is often due to provisions in their employment contracts that provide for automatic termination of the contract upon reaching this age limit (Börsch-Supan et al., 2023). For men, the regular retirement age was 65 until 2012. For women, it was 60 under certain circumstances until 2000 and was gradually raised to 65 between 2000 and 2009. However, starting in the 1980 s it was possible in West Germany to leave the labour market at the age of 58 and then retire at the age of 60 with a bridging period of two years of unemployment benefits (Buchholz et al., 2013). These measures were implemented in a period of high unemployment, also related to the labour market entry of the baby boom cohorts, and aimed at fostering early retirement. In reunified Germany, these regulations continued to exist with some adjustments and restrictions until 2007. Starting in 2012, the statutory retirement age has gradually been increasing for both women and men. It will reach 67 by 2031 and will remain at this age as long as no additional legislation to change the statutory retirement age is passed. In addition, further reforms have been implemented to prevent or limit early retirement before the regular retirement age.

### Inequalities in Employment Between Eastern and Western Germany, Men and Women, and Education Groups

The periods of high unemployment in Germany described above were characterised in particular by strong regional and age-related disparities, with major differences recorded between eastern and western Germany. At over 20 per cent, the unemployment rate in eastern Germany in the mid-2000s was twice as high as in western Germany (Federal Statistical Office of Germany, 2024d). Clear East-West-disparities can still be seen today, even if they are no longer as pronounced. Irrespective of region, various age-specific differences can be identified for individual periods. In the 1990s, unemployment was particularly high among older people, while in the 2000s it was younger people under the age of 25 who were more likely to be affected (Eurostat, 2024b). The high unemployment figures for older people in the 1990s can be explained by the above-discussed early retirement regulations that facilitated periods of unemployment right before retirement, as well as general economic conditions (Buchholz et al., 2013).

The German labour market is highly gendered. As well as having lower labour force participation rates, women work fewer hours in paid employment than men. Care responsibilities for family members are one reason for interruptions or restrictions in employment. Despite changes over time, this aspect is still strongly characterised by gender-specific differences. The reduction in employment in this context continues to be significantly higher for women than for men, although there are regional differences too. These are reflected both in labour force participation and in the number of hours worked and vary between eastern and western Germany (Müller & Fuchs, 2020, Geyer et al. 2024). The employment rates of eastern German women were already higher than those of western German women prior to the division of Germany after 1945 (Becker et al., 2020). Employment differences increased further during the division of the country and were very strong at the beginning of the 1990s. Although employment participation has converged to some degree since reunification, women in eastern Germany are still much more likely to work full-time, whereas part-time work predominates among their counterparts in western Germany, especially among mothers (Federal Statistical Office of Germany, 2024b, Barth et al., 2020, Pfahl et al., 2023). These disparities are intertwined with similar differences in attitudes towards the employment of women and mothers in particular, which is more tolerated in eastern Germany (Bozoyan & Schmiedeberg, 2024; Gambaro et al., 2024). However, here too, some convergence between eastern and western Germany has been observed (Barth et al., 2020).

As to disparities in education, Germany has a highly standardised education system in which the level of education – in particular the possession of formal educational qualifications – significantly influences labour market participation (Becker & Blossfeld, 2021; Manzoni et al., 2014). This impact is evident both when entering the labour market and throughout middle and old age. Compared with individuals possessing a higher level of education, those with lower skills experience higher unemployment rates over the course of their working lives, especially during economic crises (Reinberg & Hummel, 2005; Seibert, 2025; Weber & Weber, 2013). This is partly due to the fact that low-skilled workers are disproportionately employed in precarious, marginal or temporary jobs, often in sectors with declining employment opportunities. Additionally, they are much less likely to find new employment if they lose their jobs, particularly at an older age. Furthermore, individuals with lower levels of education face a higher risk of early retirement and disability (Fasang, 2010; Hagen et al. 2010).

### Working Life Expectancy and its Potential

WLE can be defined as the expected number of years spent being economically active or employed, either in a given age range or over the whole life course (Dudel et al., 2023; Dudel & Myrskylä, 2020; Loichinger & Weber, 2016). It can be viewed from both a cohort and a period perspective. The period perspective shows a hypothetical WLE that indicates how many years an individual would be in employment over the course of their entire life if the age-specific WLEs of the calendar year under consideration remained unchanged throughout their working life. The period WLE as specified in this paper is calculated from the sum of all 60 age-specific WLEs for the age range 15 to 74 years for a calendar year. It is comparable to long-established measures such as the period life expectancy used to sum up age-specific mortality rates in a given period or the total fertility rate based on annual age-specific birth rates. When WLE is calculated from the cohort perspective, working trajectories of actual birth cohorts are observed. However, data demands are considerably higher for the cohort perspective than for the period perspective, which is likely the main reason as to why it is rarely used (see Dudel et al., 2023 for an exception).

When calculating WLE, the literature often only takes into account whether or not a person is employed or economically active (Dudel et al., 2018; Loichinger & Weber, 2016; Tetzlaff et al., 2022). Working time, measured in hours, is rarely considered. In cases where full-time employment is standard and part-time employment is rare, this approach can be useful. In Germany, however, the consideration of weekly working hours is of central importance given that the proportion of individuals, particularly women, working part-time is notably high (Eurostat, 2024a, Federal Statistical Office of Germany, 2024a). Furthermore, our data also include information regarding potential secondary and tertiary employment in more than one job, as well as the corresponding working hours. Given that in 2022, over 4 per cent of men and more than 5 per cent of women were employed in more than one job, the impact on the calculated employment volumes is not negligible.

Compared with many other studies, our data source also allows us to examine trends in desired working hours and the resulting over- and underemployment from 2008 onwards. Based on this data, we extend the concept of the WLE to quantify unrealised WLE, and subsequently evaluate the potential WLE that could be realised.

## Data and Methods

### Data: The German Microcensus

We use data from the German Microcensus. The Microcensus is the largest annual household survey of official statistics in Germany, and has been carried out jointly by the Statistical Offices of the Federation and the Federal States since 1957. Roughly 810,000 people in around 370,000 private households and collective accommodation (approximately 1 per cent of the population) are surveyed about their working and living conditions. Participation in the survey is compulsory if a household is sampled. This makes the nonresponse rate very low, at around 5 per cent per year for the years up to 2019. Following the methodological redesign of the Microcensus in 2020, this rate temporarily increased and was most recently around 12 per cent in 2022 (Federal Statistical Office of Germany, 2023b). The key items in the questionnaire, many of which are compulsory to answer, also have a very low nonresponse rate. In contrast to many other surveys on labour market participation, the Microcensus includes data on all forms of employment (private sector workers, public sector workers, civil servants, the self-employed, etc.). Our analysis covers the years 1991 to 2022, as 1991 is the first year that provides information for eastern Germany. We include observations in our analysis that cover individuals in the age range from 15 to 74 years. Comparable information on desired working hours has only been available for the years 2008 to 2022. For an overview of the Microcensus, see Federal Statistical Office of Germany (2017, 2023b) and Hundenborn and Enderer (2019).

The variables used in our analysis are defined as follows. We consider individuals as being employed or unemployed in accordance with the conventions of the International Labour Organization (ILO). This means that individuals are regarded to be employed if they work at least one hour per week. They are categorised as unemployed if they are not employed, have actively been looking for work in the past four weeks, and are available to start work within the next two weeks. Individuals who are neither employed nor unemployed are considered to be inactive. The working hours of an individual are captured through a question asking for the typical number of hours worked in a week, including overtime. It is important to note that we also take working hours in potential second and third jobs into account. The desired number of working hours is captured by asking individuals whether they would agree to increase or decrease their working hours if their income was to increase or decrease accordingly. If they state that they are willing to modify their working hours, they are then asked to specify the extent of this change (in hours per week). This information is used to measure either over- or underemployment. Educational attainment is classified based on a coarsened version of the International Standard Classification of Education (ISCED) of 2011, using three levels: at most lower secondary education (ISCED levels 0 to 2); upper and post-secondary non-tertiary education (ISCED levels 3 to 4); and tertiary education (ISCED levels 5 to 8). Results are also stratified by gender and region (eastern vs. western Germany; Berlin is included in eastern Germany).

Our analyses of WLE for the age range 15 to 74 years are based on case numbers per year of age, ranging between 3,114 (74-year-olds in 1991) and 13,445 (52-year-olds in 2017). The number of cases is determined by the size of the population of the respective age in the respective calendar year.

### Measuring Employment Potential and Potential WLE

To measure and quantify employment potential and, subsequently, potential WLE, it is essential to first define and delineate employment potential as a concept. According to the International Labour Organization (ILO), labour force potential includes all individuals who are either employed or unemployed (see, for example, the definition of the labour force survey in (Hussmanns et al., 1990). These groups can be clearly identified and delineated based on the existing definition and the data available. However, broader or alternative conceptualisations of labour force potential may also include groups of individuals who are neither employed nor unemployed. Based on such concepts for identifying so-called hidden unemployment and hidden underemployment, individuals are included who are actively seeking employment but are not immediately available to work in the short term. It also includes individuals who are not actively seeking employment at present, for instance, because they see no prospect of securing a job under the current labour market conditions (so-called “discouraged workers”). Nonetheless, these individuals express a desire to work and are generally available for employment.

No internationally accepted or standardised definition currently exists for the concepts of hidden unemployment or hidden underemployment. Consequently, the composition of this group varies depending on the specific definitions and delimitation criteria applied (see Sengenberger, 2011; Konle-Seidl & Lüdeke, 2017). In Germany, the hidden labour force is estimated by the Federal Statistical Office and the Institute for Employment Research (IAB), among others. However, due to the utilisation of differing conceptual frameworks, the estimated size of the hidden reserve varies accordingly (see Fuchs & Weber, 2011; Rengers & Fuchs, 2022).

Given the challenges in defining and measuring hidden unemployment and hidden underemployment described above, this paper focuses solely on those individuals and their actual and desired working hours who are classified as either employed or unemployed in line with the definition of the labour force survey when measuring untapped potential. Reliable data regarding the number and socio-demographic characteristics of these groups are available for the period under review. Consequently, the definition of employment potential underlying our analyses is narrow and confined to the current labour market situation under the existing structural conditions. From this point onwards, we will refer to this as “employment potential”.

### Methods: Aggregating Individual-Level Working Trajectories

The concept of WLE describes the expected number of years spent in employment between the ages of 15 and 74 years (Loichinger & Weber, 2016). We determine the length of time spent in employment, measured in full-time equivalents, using a modified version of Sullivan’s (1971) method. This is consistent with the approach described by Dudel et al. (2023). First we set the age-specific weekly working hours of the respective employed population in relation to full-time working hours, defined as 40 h per week. The latter figure was the average weekly number of working hours for full-time employees in 2022 (Federal Statistical Office of Germany, 2023a). We use this to derive age-specific weighting factors. In order to calculate WLE, we then multiply these age-specific weighting factors by the age-specific employment rates. After that, the age-specific WLE values are aggregated for all age groups between 15 and 74 years.

To calculate lifetime unemployment, the age-specific proportion of the unemployed population is first calculated. As far as weekly working hours are concerned, it is assumed that an unemployed person would after taking up a job work the same number of hours per week as an employed person. Accordingly, the calculated, age-specific proportion of the unemployed population is multiplied by the average, age-specific weekly working hours of the employed population. The age-specific unemployment durations are then totalled over the age range of 15 to 74 years.

In order to calculate the additional employment potential due to unfulfilled working time desires, the difference between the desired and the actual weekly working time in hours is determined. This value is then divided by the number of full-time working hours (40 h) and multiplied by the age-specific employment rate. Afterwards, the age-specific values are aggregated for all age groups between 15 and 74 years. All indicators are calculated annually.

It is important to note that at younger ages, the age-specific WLE differentiated by level of education should be interpreted with caution due to issues of both over- and underestimation. This is due to the fact that education has not yet been completed in some cases at these ages and the level of education is only assigned to people from the age at which this level was attained. Accordingly, employment undertaken before this level of education was reached is assigned to the correspondingly lower level of education. For example, a university student who is rarely employed on a full-time basis would therefore be counted as having a medium level of education up to graduation. The resulting bias also has implications for interpreting lifetime WLE estimates by education.

## Results

### Trends in Working Life Expectancy

**Fig. 1:**
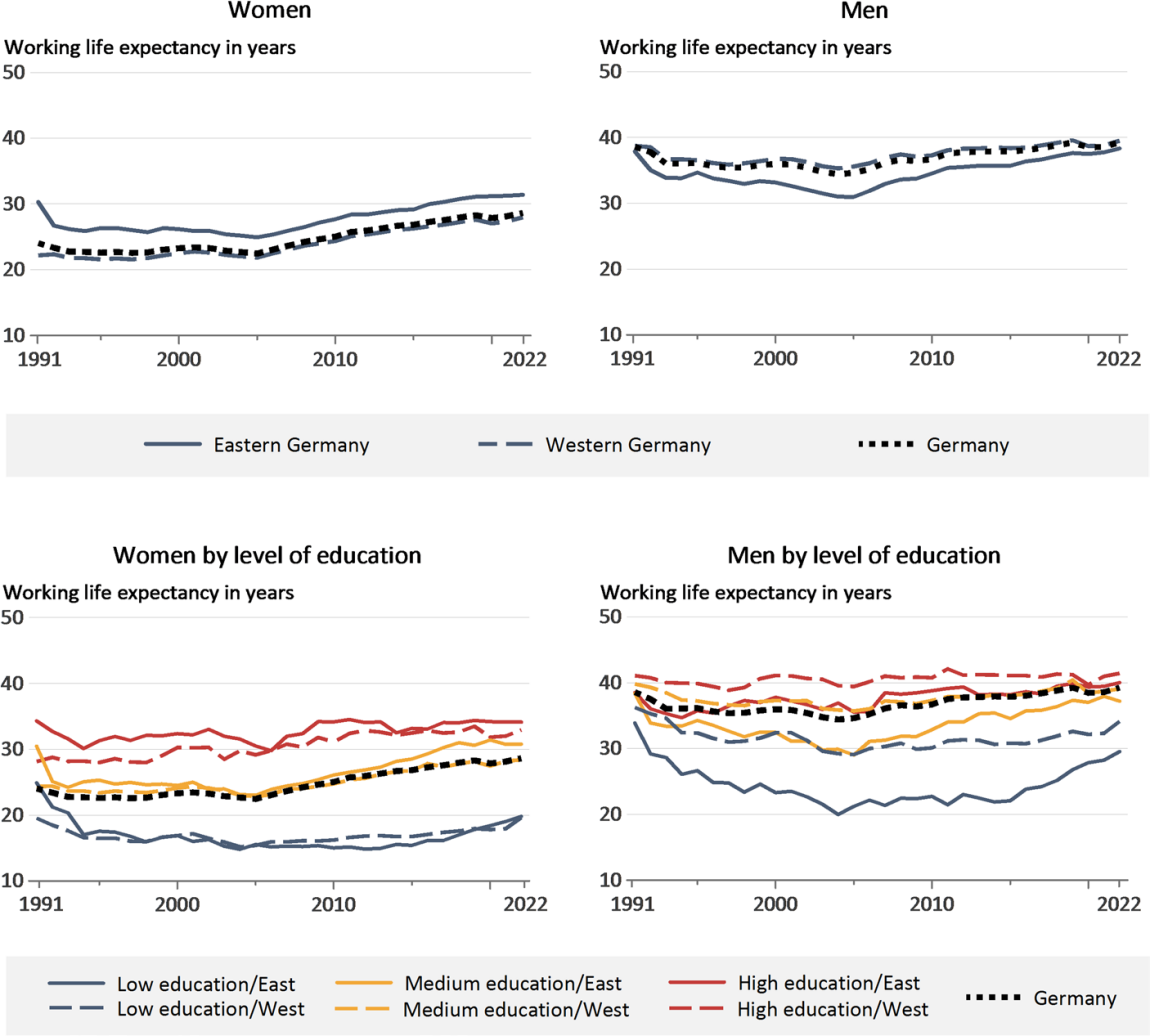
Working life expectancy (WLE, measured in full-time equivalent years) by region (eastern/western Germany), gender, and level of education according to the ISCED-2011 classification (1991–2022). Largest 95% confidence interval for WLE by region and gender (upper panels): Eastern German men in 2004 (WLE: 31.0 years; lower bound: 30.8 years; upper bound: 31.2 years). Smallest 95% confidence interval: Western German women in 2022 (WLE: 28.0 years; lower bound: 27.9 years; upper bound: 28.0 years). Largest 95% confidence interval for WLE by region, gender, and level of education (lower panels): Eastern German men with a high level of education in 1991 (WLE: 38.5 years; lower bound: 37.5 years; upper bound: 39.5 years). Smallest 95% confidence interval: Western German men with a medium level of education 1991 (WLE: 39.8 years; lower bound: 39.7 years; upper bound: 39.9 years). Source: RDC of the Federal Statistical Office and Statistical Offices of the Federal States of Germany, (2025), Microcensus 1991–2022, authors’ calculations.

The development of WLE by gender and region, and WLE by gender, region, and level of education over our observation period from 1991 to 2022 is shown in Fig. 1. The WLE values can be interpreted in the same way as period life expectancy: they represent the average number of full-time equivalent years that an individual aged 15 would spend in employment up to the age of 74 years, assuming that the age-specific employment rates and weekly working hours of the respective year remain constant throughout the entire working life. Measurement is thus in full-time equivalent years, where one year of full-time work contributes a full year, while part-time work contributes less. Due to the large sample size of the Microcensus, the confidence intervals for the WLEs are very narrow. As they would therefore be barely visible in the figures, we have chosen not to display them. Instead, selected confidence intervals are provided below Fig. 1 as illustrative examples.

The upper panels of Fig. 1 show WLE by gender and region (eastern vs. western Germany). In 1991, WLE in full-time equivalents was 24.1 years for women and 38.6 years for men. Among women, eastern Germans had a far higher WLE than their western German counterparts, with values of 30.3 years and 22.2 years respectively. By contrast, western German men had a higher WLE than eastern German men, but the differences were comparatively small, at 37.9 years (East) vs. 38.7 years (West). The levels recorded for all four groups are higher in 2022 than in 1991, but the differences are rather small among eastern German women (+ 1.1 years), western German men (+ 0.8 years), and eastern German men (+ 0.4 years). However, women in general and western German women in particular, experienced a substantial increase. At 28.0 years, the WLE for western German women in 2022 is 5.8 years higher than in 1991. The patterns for Germany as a whole are largely driven by, and follow, the trends in western Germany, as individuals in eastern Germany represent only about 20 per cent of the population.

Looking at the development year-on-year, we observe that WLE dropped in economically weak phases such as in the 1990s and mid-2000s. However, the declines varied across the different groups. The decline in the early to mid-1990s hit eastern Germany much harder, reflecting the consequences of high unemployment in the years following German reunification as a result of the economic transformation process in the former communist part of the country. A more detailed analysis of the age-specific changes is shown in Fig. A1 in the supplementary materials.

When we turn to the differentiation by level of education, the lower panels of Fig. 1 show that western German men with a high level of education record the highest WLE over the entire period under review. By contrast, the derived WLE of women with a low level of education is particularly low, both in eastern and western Germany. Comparing the two groups with the highest and lowest WLE at the end of the observation period, highly educated western German men have a WLE of 41.4 years in 2022, which is more than twice that of western German women with a low level of education (19.6 years). A comparison over time shows some divergent trends across genders, regions, and educational levels. Notably, the comparison of 1991 and 2022 shows that women and men in eastern Germany with a low level of education experienced substantial declines, with reductions of more than five and four years of WLE, respectively. However, it should be noted that such individuals in eastern Germany constitute a very small and selective group. Women in western Germany with medium and high levels of education achieved significant increases in WLE, of 4.1 and 4.8 years respectively. All other groups experienced comparatively minor changes. Additional differentiated age- and education-specific working life expectancies for 2022 can be found in the supplementary materials (Table A1).

It is relevant to note that the general trend in WLE is influenced by both education-specific WLE and the population’s educational composition. Declines in the early 1990s and mid-2000s, as well as the subsequent increase, were primarily the result of changes in WLE among those with low and medium levels of education; WLE among the highly educated changed little. Nevertheless, an increasing share of highly educated individuals, along with their higher WLE, led to a slight increase in the total WLE (for trends of the population share by level of education, see Fig. A2 in the supplementary materials).

### Unrealised Working Life Expectancy

**Fig. 2:**
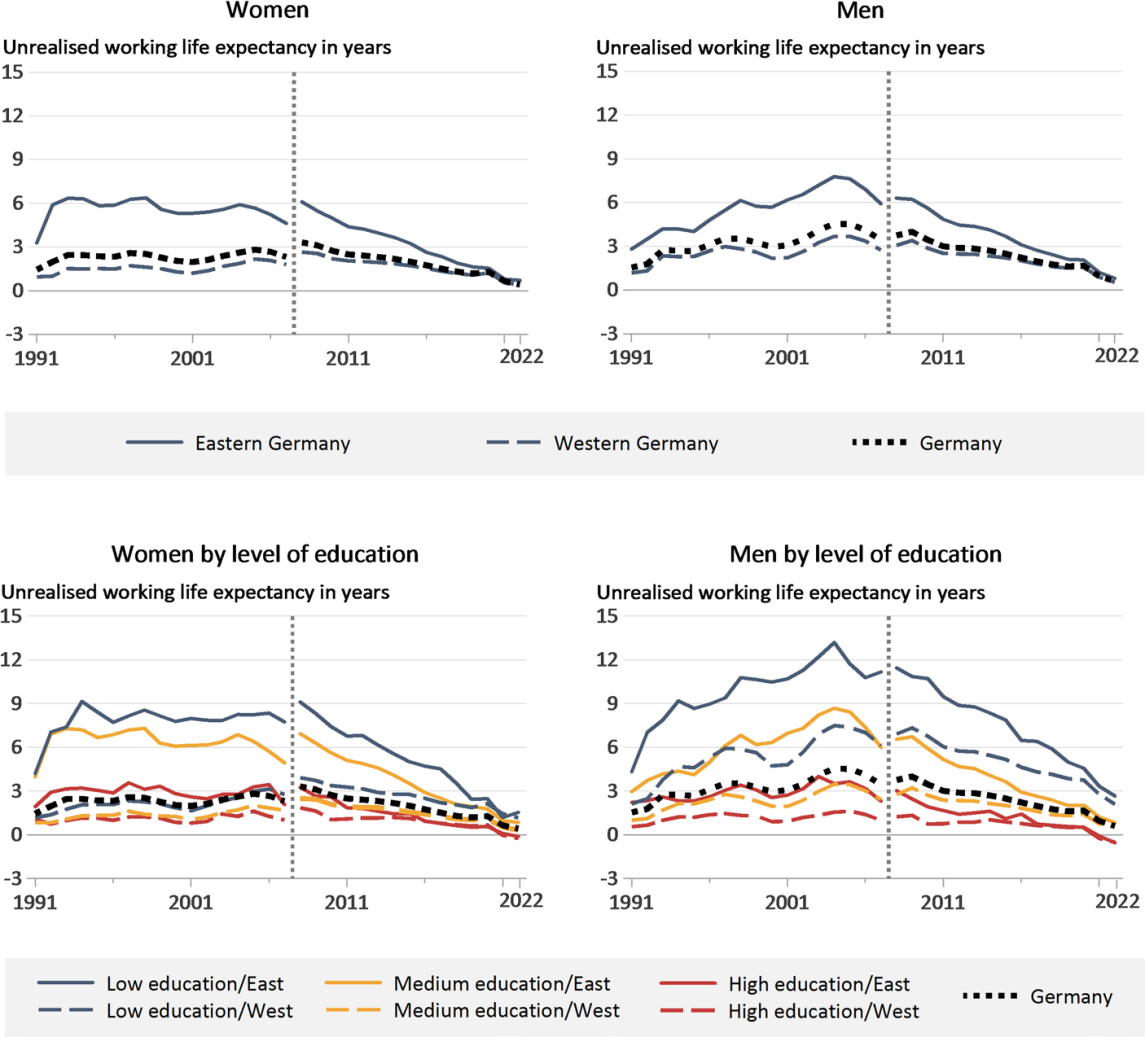
Unrealised working life expectancy (measured in full-time equivalent years) by gender, region (eastern/western Germany), and level of education according to the ISCED-2011 classification (1991–2022). The break in the figures from 2007 to 2008 is the result of data availability limitations. For the years 1991 to 2007, the unrealised working life expectancy only shows the lifetime in unemployment; for the years from 2008 onwards, it is the sum of the lifetime in unemployment and net underemployment. Source: RDC of the Federal Statistical Office and Statistical Offices of the Federal States of Germany, (2025), Microcensus 1991-2022, authors’ calculations

Trends in unrealised WLE by level of education, as measured in full-time equivalent years, are shown in Fig. 2. The unrealised WLE, figures for which have been available since 2008, is made up of the lifetimes in unemployment and net underemployment. As data on net underemployment is not available for the years prior to 2008, the unrealised WLE for the years up to 2008 shows only the lifetime in unemployment, resulting in a break in the trends shown in Fig. 2.

In the upper panel the long-term trend for all groups shows a downward trajectory, with strong outliers during the weak economic phases of the 1990s and mid-2000s. In the early 1990s, the increase was particularly strong among eastern German women, while the increase between 2000 and 2005 was greater among eastern German men. In an East-West comparison, this development in the first half of the observation period led to a divergence among men, while a convergence was observed among women from the mid-1990s onwards. Since 2005, East-West differences have narrowed significantly for both sexes. Specifically, the unrealised WLE for women was 1.4 years in 1991 (East: 3.3; West: 1.0), while it is 0.4 years in 2022 (East: 0.7; West: 0.4). For men, the unrealised WLE was 1.5 years in 1991 (East: 2.8; West: 1.2) as opposed to 0.6 years in 2022 (East: 0.8; West: 0.5). The high figures recorded for women from eastern Germany at the beginning of the 1990s were the result of changes in the eastern German labour market following the post-reunification transformation process. Employed women were more likely than men to see their jobs devalued in the adjustment process, whereas men, on average, improved their professional position (Holst & Schupp, 1995). The higher figures for eastern German men compared with eastern German women from the start of the 2000s onwards were also the due to an increasing number of the latter leaving the labour market (Lechner & Wunsch, 2009).

The differentiation by education level in the lower panels of Fig. 2 consistently shows lower unrealised WLE among the highly educated. In 2022, eastern and western German men with a high level of education have the lowest unrealised WLE (−0.5 years), while eastern German men with a low level of education have the highest unrealised WLE (2.6 years). Overall, highly educated men and women in eastern and western Germany both report a negative unrealised WLE, indicating corresponding overemployment in these groups. The disaggregation by level of education also shows that trend changes were significantly smaller for people with medium and high levels of education than for people with a low level of education, even during economically weaker phases. Overall, the current unrealised WLE offers barely any potential for further increases in lifetime employment. Results of additional analyses that looked separately at trends in the two components of unrealised WLE, unemployment and under-/overemployment and the population-level potential resulting from the unrealised WLE at the individual level, are available in the supplementary materials (Tables A2, A3, A4 and Fig. A3).

### Potential WLE and the Contributions of Realised WLE and Unrealised WLE

Potential WLE, as defined in Sect. 3.2, comprises both realised WLE and unrealised WLE. Both realised WLE and unrealised WLE, in turn, consist of two elements. Realised WLE is influenced by the employment rate and working hours, whereas unrealised WLE is based on unemployment and over-/underemployment. In terms of unrealised WLE, this definition refers to the potential of the labour force. The potential of individuals not in the labour force who could enter the labour force later or under altered conditions is not considered here. Potential WLE, therefore, indicates the maximum level of WLE for a population group that would be achieved if all unemployed individuals within the group were employed and all employed individuals could work their preferred number of hours per week under existing conditions. Fig. A4 in the supplementary materials summarises potential WLE for the individual population groups.

**Table 1:** Results of the decomposition analysis of the differences in potential working life expectancy (WLE) by gender, region (eastern/western Germany), and level of education, 2022

Gender	Region	Education level	Difference in potential WLE (in years)	Components			
				Employment	Working hours	Unemployment	Unfulfilled/Overful-filled working time desires
Men	West	High	–	Reference: 40.9 years			
		Medium	−1.3	−1.7	−0.7	+ 0.4	+ 0.6
		Low	−4.7	−6.4	−0.9	+ 1.5	+ 1.1
	East	High	−1.5	−1.0	−0.4	−0.3	+ 0.2
		Medium	−2.9	−3.4	−0.8	+ 0.6	+ 0.8
		Low	−8.7	−10.1	−1.8	+ 1.9	+ 1.3
Women	West	High	–	Reference: 32.7 years			
		Medium	−3.9	−2.5	−2.0	0.0	+ 0.5
		Low	−11.9	−10.9	−2.4	+ 0.5	+ 0.9
	East	High	+ 1.3	−1.2	+ 2.4	+ 0.1	+ 0.1
		Medium	−1.0	−2.8	+ 0.7	+ 0.3	+ 0.8
		Low	−11.3	−13.0	−0.1	+ 0.7	+ 1.1

The “Difference” column shows the differences in potential WLE between the reference group and the corresponding group (measured in full-timeequivalent years).

Source: RDC of the Federal Statistical Office and Statistical Offices of the Federal States of Germany, (2025), Microcensus 1991-2022, authors’calculations.

In order to quantify the impact of the individual components of potential WLE on the differences between the socio-economic groups, a decomposition analysis was conducted using data from 2022. Table 1 displays the results, breaking down the differences in potential WLE for the individual population groups by gender, regional differences, and level of education. The decomposition shows the contributions of the employment rate, weekly working hours, unemployment, and under- or overemployment due to unfulfilled working time desires to the overall difference.

For each component the value in Table 1 shows how much the component contributes to the total difference between groups. A negative value for a component indicates that the comparison group has a lower realised WLE or, respectively, unrealised WLE for that component than the reference group. Conversely, a positive value suggests the opposite. The sum of the contributions of the four components equals the observed difference. For instance, for the comparison between western German men with high and medium educational attainment, the total difference is −1.3 years; i.e., the medium educated have a slightly lower potential WLE compared to the highly educated. This overall difference is largely due to lower employment rates (−1.7 years) and lower working hours (−0.7 years) for the medium educated. Unemployment and underemployment, on the other hand, have positive values of + 0.4 and + 0.6 years, respectively. This indicates that these two components, in isolation, lead to a higher potential WLE for the medium educated compared to the highly educated. In total, unemployment and underemployment cancel out some of the total negative effect of employment rates and working hours, but not all of it.

For men, the biggest difference in potential WLE is −8.7 years, observed between the reference group (western German men with a high education level) and eastern German men with a low education level. This difference mainly arises from the lower employment rate among eastern German men with a low level of education, which negatively affects their WLE by −10.1 years. In contrast, this group spends 1.9 years longer in unemployment compared with the reference group. The effects of differences in weekly working hours (−1.8 years) and underemployment (+ 1.3 years) are of similar magnitudes. Among women too, differences in employment rates predominantly account for variation in potential WLE. However, in this case, the effect of differences in weekly working hours is also relatively substantial, particularly for western German women.

Tables A5a to A5c in the supplementary materials show additional decomposition analyses for the years 1991, 2001, and 2011. These tables provide results largely consistent with the findings of Table 1, with the exception of working hours. For women, working hours were relevant in all years. For men, however, working hours did play almost no role in 1991 (Table 4a); in contrast, in 2022, working hours contributed to some extent. Results for 2001 and 2011 are in between. This indicates that differences in working hours have become more relevant for men, even though their contribution is still moderate.

## Discussion

### Key Findings

Using data from the German Microcensus for the years 1991 to 2022, we analysed long-term population-level trends in the lengh of working life (working life expectancy, WLE) for the first time for Germany. In doing so, we not only accounted for variation in employment rates, but also for variation in working hours. Furthermore, we identified untapped employment potential among various subgroups in Germany (differentiated by gender, region (eastern/western Germany), and level of eduation). We calculated WLE at age 15 adjusted for working hours, and potential WLE accounting for unemployment, underemployment, and overemployment. Our analysis provides four main insights. First, most groups exhibit a U-shaped development, in which WLE in 2022 is similar to that in 1991. One exception are women in western Germany with a high or medium level of education, among whom the WLE obtained for 2022 was much higher than that for 1991. On the other hand, individuals with a low level of education record a significantly lower WLE in 2022 than in 1991. A third exception are highly educated men in western Germany, who experienced only limited fluctuations. Second, inequalities in WLE are substantial. In 2022, the last year under observation, WLE ranges from 41.4 years among highly educated males in western Germany to 19.6 years among women with a low level of education in western Germany. Third, trends in unrealised WLE mirror trends in WLE – when WLE declines, unrealised WLE increases. For more recent years, this implies a decline in unrealised WLE. On the other hand, potential WLE, the sum of realised and unrealised WLE, remains relatively constant over the entire period under review. Fourth, inequalities in unrealised WLE remain substantial despite reductions in the last two decades. Individuals with a low education level continue to display a considerable degree of unrealised potential, while for highly educated individuals, virtually no unrealised potential remains.

After declining significantly up to 2005, the WLE of the population as a whole increased. Currently, it is only slightly above the level registered in 1991. The steady increase since 2005 is consistent with trends in employment rates for Germany (Federal Statistical Office of Germany, 2024b). This development can likely be attributed primarily to a prolonged period of robust economic growth accompanied by corresponding trends in the labour market. This is particularly true for women: among women in western Germany with a high and medium level of education, the four or five years gained in WLE are primarily attributable to a significant rise in the employment rate of nearly 19% points (Federal Statistical Office of Germany, 2024b). Additionally, the increase in WLE might be linked with numerous labour market and pension reforms, especially for women and older individuals (Hess et al., 2020). However, our analysis does not allow us to look into causal effects of these reforms. Nevertheless, despite these gains, a pronounced disparity remains between western German women and their male counterparts. In 2022, the WLE of western German women was 28.0 years, while western German men could expect 39.5 years in employment. This substantial difference is not particularly surprising given the high proportion of women who work part-time (Federal Statistical Office of Germany, 2024e). In contrast, the difference between eastern German women and men is only 6.9 years compared with 11.5 years in western Germany. When weekly working hours are discounted, the WLE difference in western Germany between men and women amounts to 4.7 years. As expected, these disparities in WLE are mainly attributable to life phases between the ages of 30 and 44, as well as 45 and 59 – periods typically associated with family formation, child-rearing, or caring for relatives, during which women in Germany tend to reduce their employment participation significantly (Fasang et al., 2012).

Disparities between the considered subgroups vary over time. The biggest disparities were recorded when WLE was lowest in the mid-2000s. Stark disparities exist most notably between men and women, and between those with high and medium education levels on the one hand versus those with a low education level on the other. The significant increase in WLE resulting from a rise in women’s participation in employment, especially in western Germany, has reduced the gap between women in eastern and western Germany to a similar extent as the overall gender gap between women and men. When comparing the educational groups, a divergence is particularly evident among women, between those with low education and those with medium and high education levels.

A direct comparison of our findings on WLE with results for other countries is difficult, as other studies usually do not adjust for working hours and often cover a different age range. For instance, Junna et al. (2024) derived WLE for Finnish men and women based on the primary activity status, not adjusting for working hours, and covering people aged 25 and older. They report that in 2020, Finnish men had at age 25 a WLE of 28 years, while for Finnish women the figure was 27 years. Using a similar setup with the Microcensus – i.e. not adjusting for working hours and covering people aged 25 and older –we find a WLE at age 25 of 35 years for German men and 32 years for German women for 2020. The trends in WLE for Finland and the education-specific differences are largely comparable with those observed in Germany. In contrast, the disparities between women and men in Germany are, as expected, significantly greater. Dudel et al. (2018) analysed WLE for Spain for the years 2004 to 2013, once again not accounting for working hours. While WLE in Spain was slightly higher than the German level from 2004 to 2007 for both women and men, it declined rapidly in the subsequent years of the recession that severely impact the Spanish labour market. This decrease in the Spanish WLE was much more pronounced compared with trends in Germany during the crisis years of the early 1990s and mid-2000s.

The measure of “duration of working life” as reported by Eurostat closely resembles the concept of WLE; however, it does not account for working hours, whereas periods of unemployment are included in the duration of working life. Consequently, our WLE values for the year 2022 are, as expected, lower than those reported by Eurostat, particularly among women (by approximately 8.7 years). When applying the same delineation and incorporating lifetime in unemployment, our WLE values differ only marginally from those provided by Eurostat.

The trends in untapped employment potential, which we measure as unrealised WLE, largely mirror the observed WLE trends. In particular, unrealised WLE has decreased significantly across all groups over the past decade. This trend is attributable to both declining unemployment and reductions in underemployment, as well as to increases in overemployment. The reduction in the duration of unemployment, like the increase in employment and WLE, occurred in a period of robust economic performance. This economic condition might have fostered this trend. Furthermore, demographic developments – most notably the decline in the working-age population – may also have contributed to this trend by increasingly enabling less qualified individuals to secure employment.

Despite the overall decline in unrealised WLE, significant disparities remain, particularly across different educational groups. For 2022, highly educated individuals exhibit a negative potential owing to very low unemployment rates and increasing overemployment. Conversely, among less educated persons, unrealised WLE remains over one year for men and more than two years for women. In 2022, unrealised WLE is the result almost exclusively of unemployment, as most groups no longer experience underemployment due to unfulfilled working time desires.

As with results on WLE in general, comparisons between unrealised WLE utilised in our study and results from other countries are not straightforward, since other studies that exist generally do not account for under- and overemployment resulting from individuals’ working time desires. However, comparisons of the partial component of lifetime in unemployment are feasible. For instance, it is evident that the lifetime in unemployment in Germany in 2020 is significantly lower than in Finland (Junna et al., 2024). Specifically, the life expectancy in unemployment for both women and men in Finland is three years, whereas in Germany it is 0.9 years for women and 1.3 years for men. Compared with Spain, which was examined by Dudel et al. (2018) for the years 2004 to 2013, the lifetime in unemployment in Germany between 2004 and 2006 was approximately twice as high as in Spain, with four to five years for men. Conversely, there were minimal differences for women. In subsequent years, the lifetime in unemployment increased in Spain, whereas it decreased in Germany.

The sum of WLE and unrealised WLE, which we have defined here as potential WLE, exhibits only minimal fluctuations over the observation period. Notably, an almost stable trend can be observed among men over the thirty-year span, and there is virtually no variation at all between the levels for men in eastern and western Germany. This could indicate that, even in times of crisis, men in Germany generally remain within the labour force. For women, an East-West convergence is evident over time, characterised by a slight decline among eastern German women and a significant increase in potential WLE among western German women.

### Methodological Considerations

Our findings show that educational inequalities in WLE from age 15 to age 74 are substantial. However, educational attainment is not fixed at age 15. Individuals may move between educational groups, while our calculations represent snapshots in time. For instance, in the Microcensus, university students are generally categorised as having a medium level of education until they complete their degree. Therefore, their employment while they studied is included in the calculations as employment of people with a medium level of education. The same applies to individuals with a medium level of education, whose employment up to achieving a medium-level qualification is treated as employment of people with a low level of education. Consequently, the WLE for the presented educational groups may include both under- and overestimations. A precise quantification is not possible with the available data and varying educational paths. In robustness analyses excluding students, the WLE for those with completed secondary education was 0.8–1.8 years higher for men and 0.7–1.2 years higher for women, depending on the year (0.8 and 0.7 for 2022). The underestimation for persons with a medium education arises from students’ lower employment rates and weekly hours. However, the actual underestimation for this group is likely lower than these figures suggest, since not all students complete their studies, and thus stay in medium education.

Our measurement of potential WLE builds on unemployment and under-/overemployment, where the latter are based on preferred working hours. This measurement has limitations. Specifically, we do not include individuals who are neither working nor looking for work, but who perhaps would have been looking for work given better economic conditions and a better labour market situation. Moreover, preferred working hours are also driven by current labour market conditions; for instance, people for whom mainly part-time jobs are available might adjust their preferences accordingly, even though they would prefer a full-time job if such a job were more easily attainable.

We employ Sullivan’s method, which is a life table application and uses a period perspective. Our results, similar to conventional calculations of life expectancy, therefore show what the outcome of a hypothetical cohort would be if the existing conditions in a given period remained the same throughout the entire working life phase. Our results may therefore not be transferable to real cohorts, or only to a limited extent (Leinonen et al., 2018). Concretely, longer-term economic downturns in the future could lead to real cohorts realizing lower WLEs than what the period perspective shows. On the other hand, continued higher employment participation at older ages could result in a higher realized WLE of younger cohorts than the WLE depicted from the period perspective. Moreover, in our analyses, we do not control for mortality as would typically be done when applying Sullivan’s method, as no mortality data differentiated by educational level are available for Germany. However, as employment and unemployment rates are very low in the highest age groups in which mortality has a significant impact, it can be assumed that this limitation has only a very minor influence on the results, as has been shown in comparable studies (Dudel et al., 2023).

## Conclusion and Outlook

Our results show that previously untapped labour market potential in Germany is increasingly exploited. In the context of continued labour market and pension reforms aimed at extending working lives, this can be seen as a success. At the same time, the level of WLE today is only slightly higher than it was at the beginning of the 1990s. Moreover, the remaining employment potential is largely limited to individuals with a low level of education within the younger and middle-aged groups. This is a key insight for debates surrounding the financing of social security systems and the labour shortage in Germany, which often focus exclusively on extending working life and on older individuals. This insight also highlights the usefulness of WLE and related measures.

To utilise this remaining potential, efforts should be directed towards education and training of the individuals making up the group in question. Additionally, further work is required in order to unlock new potential. In particular, the comparatively low number of hours worked by women in western Germany should be highlighted. The number of weekly hours of gainful employment that mothers consider ideal exceeds the potential quantified here (Bujard & Kleinschrot, 2024). However, these are not readily available and attaining them requires changes in the framework that shapes individuals’ employment decisions. Conversely, it is important to consider that the current labour force, as well as the calculated WLEs and potentials based on it, include groups who would prefer to reduce their working hours or cease working – particularly older persons – but do not do so due to financial considerations.

It is unfortunate that Germany seems to be one of the few countries that enables population-level WLE and unrealised WLE to be derived while taking realised working hours and working hour preferences into account. We believe that our study demonstrates the benefits of considering these aspects. As a result, we hope that our study sets standards for future data collections and studies in other countries aiming to assess WLE and untapped employment potential.

## Supplementary Information

Below is the link to the electronic supplementary material.

Supplementary Material 1

## Data Availability

The data supporting the findings of this study are available from the Research Data Centre of the Federal Statistical Office and the Statistical Offices of the Federal States of Germany, subject to restrictions and fees. https://www.forschungsdatenzentrum.de/de/haushalte/mikrozensus.
