# Supplementary material for: Trends in Working Life Expectancy and Untapped Employment Potential in an Ageing Population: The Case of Germany
Source: Eur J Popul. 2026 Apr 25;42(1):14. doi: 10.1007/s10680-026-09773-x (PMC13133311; doi:10.1007/s10680-026-09773-x)
Supplement: Supplementary file 1 — Supplementary Material 1 [file 10680_2026_9773_MOESM1_ESM.docx]

**Supplementary Materials: Trends in Working Life Expectancy and Untapped Employment Potential in an Ageing Population: The Case of Germany**

Harun Sulak, Christian Dudel, Elke Loichinger, Sebastian Klüsener

April 2026

Figure A1 illustrates age-specific WLE differences by comparing 2022 with 1991. A negative value indicates that the contribution of working time, measured in full-time hours, to WLE in the respective age group is lower in 2022 than in 1991, whereas a positive value signifies a higher contribution in 2022 relative to 1991.

Among men in western Germany, a decline in WLE is observed between the two reference years in the age groups from 15 to 51 years. This is in contrast to an increase in subsequent age groups. Overall, this results in an increase of 0.8 years in WLE.

**Figure A1: Full-time equivalent age-specific working life expectancy (WLE) (2022 vs. 1991)**

Source: RDC of the Federal Statistical Office and the Statistical Offices of the Federal States of Germany, Microcensus 1991-2022, authors’ calculations.

Among western German women, only the values in the age groups 15 to 25 years declined; in other age groups, there were notable increases in some cases. Overall, women in western Germany experienced the biggest increase, with WLE rising by 5.8 years between 1991 and 2022.

Among eastern German men, the decline observed between ages 15 and 49, followed by an increase in later years, results in an overall increase of 0.4 years. For eastern German women, the values decreased relatively sharply up to age 51. However, due to significantly higher employment rates among those aged 52 and older, an overall increase of 1.1 years can be observed for this group.

**Figure A2: Change in the composition of the population aged 15 to 74 years by level of education (1991-2022)**

Source: RDC of the Federal Statistical Office and the Statistical Offices of the Federal States of Germany, Microcensus 1991-2022, authors’ calculations.

**Table A1: Remaining working life expectancy (WLE) by age, gender, and level of education in Germany (2022) (measured in full-time equivalent years)**

|  | **Women** | | | |  | **Men** | | | |
| --- | --- | --- | --- | --- | --- | --- | --- | --- | --- |
| **Age** | **Total** | **Low** | **Medium** | **High** |  | **Total** | **Low** | **Medium** | **High** |
| **15** | 28.6 | 19.6 | 29.0 | 33.6 |  | 39.3 | 33.6 | 38.8 | 41.4 |
| **16** | 28.6 | 19.6 | 29.0 | 33.6 |  | 39.2 | 33.5 | 38.8 | 41.4 |
| **17** | 28.4 | 19.4 | 28.7 | 33.6 |  | 39.0 | 33.2 | 38.4 | 41.4 |
| **18** | 28.2 | 19.2 | 28.4 | 33.6 |  | 38.6 | 32.9 | 38.1 | 41.4 |
| **19** | 27.8 | 18.8 | 28.0 | 32.7 |  | 38.1 | 32.4 | 37.6 | 40.9 |
| **20** | 27.3 | 18.3 | 27.6 | 32.1 |  | 37.6 | 31.7 | 37.2 | 40.4 |
| **21** | 26.8 | 17.7 | 27.2 | 31.5 |  | 37.0 | 31.1 | 36.6 | 39.8 |
| **22** | 26.3 | 17.1 | 26.6 | 31.0 |  | 36.4 | 30.4 | 36.0 | 39.2 |
| **23** | 25.7 | 16.6 | 26.1 | 30.4 |  | 35.7 | 29.8 | 35.4 | 38.6 |
| **24** | 25.1 | 16.2 | 25.4 | 29.7 |  | 35.0 | 29.1 | 34.7 | 37.9 |
| **25** | 24.5 | 15.7 | 24.8 | 29.0 |  | 34.3 | 28.4 | 34.0 | 37.2 |
| **26** | 23.8 | 15.3 | 24.1 | 28.3 |  | 33.6 | 27.7 | 33.2 | 36.4 |
| **27** | 23.1 | 14.9 | 23.4 | 27.5 |  | 32.8 | 27.0 | 32.4 | 35.6 |
| **28** | 22.4 | 14.5 | 22.7 | 26.7 |  | 31.9 | 26.3 | 31.5 | 34.7 |
| **29** | 21.7 | 14.2 | 22.0 | 25.9 |  | 31.1 | 25.6 | 30.7 | 33.8 |
| **30** | 21.1 | 13.8 | 21.3 | 25.1 |  | 30.2 | 24.9 | 29.8 | 32.8 |
| **31** | 20.4 | 13.4 | 20.6 | 24.3 |  | 29.3 | 24.1 | 28.9 | 31.9 |
| **32** | 19.7 | 13.1 | 20.0 | 23.5 |  | 28.4 | 23.4 | 28.0 | 30.9 |
| **33** | 19.1 | 12.7 | 19.3 | 22.8 |  | 27.5 | 22.7 | 27.1 | 30.0 |
| **34** | 18.4 | 12.3 | 18.7 | 22.1 |  | 26.6 | 22.0 | 26.2 | 29.0 |
| **35** | 17.8 | 12.0 | 18.0 | 21.3 |  | 25.7 | 21.2 | 25.3 | 28.1 |
| **36** | 17.2 | 11.6 | 17.4 | 20.6 |  | 24.8 | 20.4 | 24.4 | 27.1 |
| **37** | 16.6 | 11.3 | 16.8 | 19.9 |  | 23.9 | 19.7 | 23.5 | 26.2 |
| **38** | 16.0 | 10.9 | 16.1 | 19.2 |  | 23.0 | 18.9 | 22.5 | 25.2 |
| **39** | 15.4 | 10.5 | 15.5 | 18.5 |  | 22.1 | 18.2 | 21.6 | 24.2 |
| **40** | 14.7 | 10.1 | 14.8 | 17.9 |  | 21.2 | 17.4 | 20.7 | 23.3 |
| **41** | 14.1 | 9.8 | 14.2 | 17.2 |  | 20.3 | 16.7 | 19.8 | 22.4 |
| **42** | 13.5 | 9.4 | 13.5 | 16.5 |  | 19.4 | 16.0 | 18.9 | 21.4 |
| **43** | 12.9 | 9.0 | 12.9 | 15.7 |  | 18.5 | 15.2 | 17.9 | 20.4 |
| **44** | 12.2 | 8.5 | 12.2 | 15.0 |  | 17.5 | 14.4 | 17.0 | 19.5 |
| **45** | 11.6 | 8.1 | 11.5 | 14.3 |  | 16.6 | 13.7 | 16.1 | 18.5 |
| **46** | 10.9 | 7.6 | 10.8 | 13.5 |  | 15.7 | 12.9 | 15.2 | 17.5 |
| **47** | 10.2 | 7.1 | 10.2 | 12.8 |  | 14.8 | 12.2 | 14.3 | 16.6 |
| **48** | 9.6 | 6.7 | 9.5 | 12.0 |  | 13.9 | 11.4 | 13.4 | 15.6 |
| **49** | 8.9 | 6.2 | 8.8 | 11.2 |  | 13.0 | 10.6 | 12.5 | 14.7 |
| **50** | 8.3 | 5.8 | 8.1 | 10.5 |  | 12.1 | 9.8 | 11.6 | 13.7 |
| **51** | 7.6 | 5.3 | 7.5 | 9.7 |  | 11.2 | 9.1 | 10.6 | 12.7 |
| **52** | 6.9 | 4.8 | 6.8 | 8.9 |  | 10.3 | 8.3 | 9.7 | 11.8 |
| **53** | 6.3 | 4.4 | 6.1 | 8.2 |  | 9.4 | 7.6 | 8.9 | 10.8 |
| **54** | 5.6 | 3.9 | 5.5 | 7.4 |  | 8.5 | 6.8 | 8.0 | 9.9 |
| **55** | 5.0 | 3.5 | 4.8 | 6.6 |  | 7.6 | 6.1 | 7.1 | 8.9 |
| **56** | 4.4 | 3.0 | 4.2 | 5.9 |  | 6.8 | 5.4 | 6.2 | 8.0 |
| **57** | 3.8 | 2.6 | 3.6 | 5.1 |  | 5.9 | 4.7 | 5.4 | 7.1 |
| **58** | 3.2 | 2.2 | 3.0 | 4.4 |  | 5.1 | 4.0 | 4.6 | 6.2 |
| **59** | 2.6 | 1.8 | 2.5 | 3.6 |  | 4.2 | 3.4 | 3.8 | 5.3 |
| **60** | 2.1 | 1.4 | 1.9 | 2.9 |  | 3.4 | 2.7 | 3.0 | 4.4 |
| **61** | 1.5 | 1.1 | 1.4 | 2.2 |  | 2.7 | 2.1 | 2.3 | 3.5 |
| **62** | 1.1 | 0.8 | 0.9 | 1.6 |  | 2.0 | 1.6 | 1.6 | 2.7 |
| **63** | 0.7 | 0.5 | 0.6 | 1.1 |  | 1.4 | 1.1 | 1.0 | 2.0 |
| **64** | 0.5 | 0.3 | 0.4 | 0.7 |  | 1.0 | 0.8 | 0.8 | 1.5 |
| **65** | 0.3 | 0.2 | 0.3 | 0.5 |  | 0.7 | 0.5 | 0.5 | 1.1 |
| **66** | 0.2 | 0.2 | 0.2 | 0.4 |  | 0.6 | 0.4 | 0.4 | 0.9 |
| **67** | 0.2 | 0.1 | 0.2 | 0.3 |  | 0.5 | 0.3 | 0.3 | 0.7 |
| **68** | 0.1 | 0.1 | 0.1 | 0.2 |  | 0.4 | 0.2 | 0.3 | 0.5 |
| **69** | 0.1 | 0.1 | 0.1 | 0.2 |  | 0.3 | 0.2 | 0.2 | 0.4 |
| **70** | 0.1 | 0.1 | 0.1 | 0.1 |  | 0.2 | 0.1 | 0.2 | 0.3 |
| **71** | 0.0 | 0.0 | 0.0 | 0.1 |  | 0.2 | 0.1 | 0.1 | 0.2 |
| **72** | 0.0 | 0.0 | 0.0 | 0.0 |  | 0.1 | 0.1 | 0.1 | 0.1 |
| **73** | 0.0 | 0.0 | 0.0 | 0.0 |  | 0.1 | 0.0 | 0.0 | 0.1 |
| **74** | 0.0 | 0.0 | 0.0 | 0.0 |  | 0.0 | 0.0 | 0.0 | 0.0 |

Source: RDC of the Federal Statistical Office and the Statistical Offices of the Federal States of Germany, Microcensus 2022, authors’ calculations.

**Age-specific lifetime unemployment and unfulfilled working time desires**

Table A2 shows lifetime unemployment and the resulting employment potential by age group for selected years. 1991 and 2022 mark the beginning and end of our observation period, while 2008 is the first year for which we have available data on working hour preferences. The potential due to unemployment peaked in 2005, with notably high values for women and men in eastern Germany. The decline observed since then has been particularly pronounced in the age groups 30-44 and 45-59 years, among both men and women. In 2022, the potential in each of these age groups is only around a quarter of the 2005 figure, with the sharpest decline occurring among eastern German men in the age group 45-59 years (from 2.7 years to 0.3 years). In contrast, the declines in the age group 15-29 years were comparatively low across all population groups and the figures even stagnated in some cases.

**Table A2: Lifetime unemployment (measured in full-time equivalent years) by gender, region (eastern/western Germany), and age group (1991, 2005, 2008 and 2022)**

|  | **Age group** | **1991** | **2005** | **2008** | **2022** |
| --- | --- | --- | --- | --- | --- |
| **Men (East)** | 15 - 29 | 0.9 | 1.1 | 1.4 | 0.5 |
|  | 30 - 44 | 0.9 | 2.5 | 1.7 | 0.5 |
|  | 45 - 59 | 0.8 | 2.7 | 1.8 | 0.3 |
|  | 60 - 74 | 0.1 | 1.4 | 0.3 | 0.1 |
|  | 15 - 74 | 2.8 | 7.6 | 5.1 | 1.4 |
| **Men (West)** | 15 - 29 | 0.3 | 0.7 | 0.7 | 0.5 |
|  | 30 - 44 | 0.4 | 1.3 | 0.8 | 0.4 |
|  | 45 - 59 | 0.4 | 1.1 | 0.7 | 0.3 |
|  | 60 - 74 | 0.1 | 0.6 | 0.2 | 0.1 |
|  | 15 - 74 | 1.2 | 3.7 | 2.4 | 1.3 |
| **Women (East)** | 15 - 29 | 1.1 | 0.8 | 1.0 | 0.4 |
|  | 30 - 44 | 1.2 | 1.8 | 1.3 | 0.4 |
|  | 45 - 59 | 0.9 | 2.1 | 1.5 | 0.2 |
|  | 60 - 74 | 0.0 | 1.0 | 0.1 | 0.1 |
|  | 15 - 74 | 3.3 | 5.7 | 4.0 | 1.1 |
| **Women (West)** | 15 - 29 | 0.3 | 0.5 | 0.5 | 0.3 |
|  | 30 - 44 | 0.3 | 0.7 | 0.5 | 0.3 |
|  | 45 - 59 | 0.3 | 0.7 | 0.5 | 0.2 |
|  | 60 - 74 | 0.0 | 0.3 | 0.1 | 0.1 |
|  | 15 - 74 | 1.0 | 2.2 | 1.6 | 0.8 |
| **Men (Total)** | 15 - 29 | 0.4 | 0.8 | 0.9 | 0.5 |
|  | 30 - 44 | 0.5 | 1.5 | 0.9 | 0.4 |
|  | 45 - 59 | 0.5 | 1.5 | 1.0 | 0.3 |
|  | 60 - 74 | 0.1 | 0.8 | 0.2 | 0.1 |
|  | 15 - 74 | 1.5 | 4.5 | 3.0 | 1.4 |
| **Women (Total)** | 15 - 29 | 0.5 | 0.5 | 0.6 | 0.3 |
|  | 30 - 44 | 0.5 | 0.9 | 0.6 | 0.3 |
|  | 45 - 59 | 0.5 | 0.9 | 0.7 | 0.2 |
|  | 60 - 74 | 0.0 | 0.4 | 0.1 | 0.1 |
|  | 15 - 74 | 1.4 | 2.8 | 2.0 | 0.9 |

Source: RDC of the Federal Statistical Office and the Statistical Offices of the Federal States of Germany, Microcensus 1991-2022, authors’ calculations.

**Table A3: Difference between desired and actual working life expectancy (measured in full-time equivalent years) by gender, region (eastern/western Germany), and age group (2008, 2015 and 2022)**

|  | **Age group** | **2008** | **2015** | **2022** |
| --- | --- | --- | --- | --- |
| **Men (East)** | 15 - 29 | 0.3 | 0.2 | 0.0 |
|  | 30 - 44 | 0.4 | 0.3 | -0.3 |
|  | 45 - 59 | 0.4 | 0.2 | -0.2 |
|  | 60 - 74 | 0.0 | 0.1 | -0.1 |
|  | 15 - 74 | 1.2 | 0.7 | -0.6 |
| **Men (West)** | 15 - 29 | 0.3 | 0.2 | -0.1 |
|  | 30 - 44 | 0.3 | 0.2 | -0.3 |
|  | 45 - 59 | 0.1 | 0.1 | -0.3 |
|  | 60 - 74 | 0.0 | 0.0 | -0.1 |
|  | 15 - 74 | 0.7 | 0.4 | -0.8 |
| **Women (East)** | 15 - 29 | 0.4 | 0.2 | 0.0 |
|  | 30 - 44 | 0.8 | 0.5 | -0.2 |
|  | 45 - 59 | 0.9 | 0.4 | -0.1 |
|  | 60 - 74 | 0.0 | 0.1 | 0.0 |
|  | 15 - 74 | 2.1 | 1.2 | -0.4 |
| **Women (West)** | 15 - 29 | 0.3 | 0.2 | -0.1 |
|  | 30 - 44 | 0.4 | 0.3 | -0.2 |
|  | 45 - 59 | 0.4 | 0.2 | -0.1 |
|  | 60 - 74 | 0.0 | 0.0 | 0.0 |
|  | 15 - 74 | 1.1 | 0.7 | -0.4 |
| **Men (Total)** | 15 - 29 | 0.3 | 0.2 | -0.1 |
|  | 30 - 44 | 0.3 | 0.2 | -0.3 |
|  | 45 - 59 | 0.2 | 0.1 | -0.3 |
|  | 60 - 74 | 0.0 | 0.0 | -0.1 |
|  | 15 - 74 | 0.8 | 0.5 | -0.8 |
| **Women (Total)** | 15 - 29 | 0.3 | 0.2 | -0.1 |
|  | 30 - 44 | 0.5 | 0.3 | -0.2 |
|  | 45 - 59 | 0.5 | 0.3 | -0.1 |
|  | 60 - 74 | 0.0 | 0.0 | 0.0 |
|  | 15 - 74 | 1.3 | 0.8 | -0.4 |

Source: RDC of the Federal Statistical Office and the Statistical Offices of the Federal States of Germany, Microcensus 2008-2022, authors’ calculations.

Table A3 shows the differences in the development of unfulfilled working time desires measured in WLE for the start (2008), middle (2015), and end (2022) of the observation period for which they were available. These displayed a declining trend in both eastern and western Germany and for both genders in almost all age groups. Differences can be seen in the magnitude of the decline. At the start of the observation period, women aged 30-44 and 45-59 years had the highest levels of unfulfilled WLE resulting from working hours. In 2008, eastern German women aged 45-59 years had the highest unfulfilled WLE of 0.9 years. In contrast, unfulfilled WLE desires were generally low in the age group 60-74 years, both in eastern and western Germany as well as among men and women. In 2022, the unfulfilled WLE desires are negative for most population groups, resulting from increasing overemployment.

**Table A4: Employment potential in full-time equivalents due to unemployed and under-/overemployed persons aged 15-74 years, by gender, region (eastern/western Germany), and level of education (2022, in thousands)**

|  | **Underemployed** | **Unemployed** | **Unrealised employment potential** |
| --- | --- | --- | --- |
| West: men - low education | -16 | 226 | 210 |
| West: men - medium education | -144 | 241 | 97 |
| West: men - high education | -206 | 112 | -94 |
| West: men - total | -367 | 583 | 216 |
|  |  |  |  |
| East: men - low education | 1 | 42 | 43 |
| East: men - medium education | -31 | 80 | 49 |
| East: men - high education | -37 | 18 | -19 |
| East: men - total | -67 | 141 | 74 |
|  |  |  |  |
| **Germany: men - total** | **-434** | **724** | **290** |
|  |  |  |  |
| West: women - low education | 3 | 107 | 110 |
| West: women - medium education | -82 | 147 | 65 |
| West: women - high education | -118 | 84 | -34 |
| West: women - total | -197 | 343 | 146 |
|  |  |  |  |
| East: women - low education | 2 | 20 | 22 |
| East: women - medium education | -8 | 55 | 47 |
| East: women - high education | -32 | 25 | -7 |
| East: women - total | -38 | 101 | 63 |
|  |  |  |  |
| **Germany: women - total** | **-235** | **443** | **208** |
|  |  |  |  |
| West: low education | -14 | 333 | 319 |
| West: medium education | -226 | 389 | 163 |
| West: high education | -324 | 196 | -128 |
|  |  |  |  |
| East: low education | 3 | 62 | 65 |
| East: medium education | -39 | 135 | 96 |
| East: high education | -69 | 43 | -26 |
|  |  |  |  |
| **West: total** | **-564** | **926** | **362** |
| **East: total** | **-104** | **242** | **138** |
| **Germany: total** | **-668** | **1,168** | **500** |

Source: RDC of the Federal Statistical Office and the Statistical Offices of the Federal States of Germany, Microcensus 2022, authors’ calculations.

**Potential resulting from unfulfilled employment desires, measured in full-time jobs**

The results on lifetime unemployment and unfulfilled WLE due to unfulfilled working time desires allow us to quantify the total untapped employment potential from these two sources, measured in full-time jobs. Figure A3 shows this potential for the period from 1991 to 2022, although data constraints mean that working time desires are only considered for the years 2008 to 2022.

While the additional employment potential, based on our definition of potential, was 1.7 million full-time jobs in 1991, this had risen to 4.1 million full-time jobs by 2005 (in each case without taking working time desires into account). In 2008, the first year in which the total potential of unfulfilled employment desires including unemployment and working time desires could be estimated, this figure was 3.92 million. Since then, there has been a steady decline in total untapped potential. Employment potential due to unfulfilled working-time desires actually started to turn negative in 2020 and, by 2022, shows a negative figure for women and an even more negative figure for men. However, the unrealised employment potential in full-time equivalents is still positive in 2022 due to the untapped potential resulting from unemployment.

Broken down by gender and region, the untapped potential declined to 216,000 jobs among western German men and 146,000 among western German women by 2022. For eastern Germany, the figure is 74,000 full-time jobs among men and 63,000 full-time jobs among women. Differentiated by level of education, it can be seen that the potential primarily consists of people with medium and low levels of education (see Table A4). In contrast, the untapped potential among people with a high level of education is negative due to overemployment. In terms of numbers, the greatest untapped potential is found among men with a low level of education. It amounts to 253,000 full-time jobs (including self-employment).

**Figure A3: Unrealised employment potential in full-time equivalents, due to unemployed and under-/overemployed* persons aged 15-74 years (1991-2022)**

* Data on underemployment and overemployment is only available for the years 2008 to 2022.

Source: RDC of the Federal Statistical Office and the Statistical Offices of the Federal States of Germany, Microcensus 1991-2022, authors’ calculations.

**Figure A4: Potential WLE by gender, region (eastern/western Germany), and level of education according to the ISCED-2011 classification (1991-2022)**

The break in the figures from 2007 to 2008 is the result of limited data due to differences in their availability. For the years 1991 to 2007, the potential working life expectancy does not include periods of underemployment due to unrealised working time desires.

Source: RDC of the Federal Statistical Office and the Statistical Offices of the Federal States of Germany, Microcensus 1991-2022, authors’ calculations.

The illustration of the potential WLE in Figure A4 shows that this was significantly less subject to fluctuations than WLE and unrealised WLE over the entire observation period of more than 30 years, only changing slightly in terms of amount. Bigger changes occurred among western German women, who have seen an increase over time, and among those with a low level of education, who for their part have seen a decrease in potential WLE.

**Table A5a: Results of the decomposition analysis of the differences in potential working life expectancy (WLE) by gender, region (eastern/western Germany), and level of education, 1991**

| **Gender** | **Region** | **Education level** | **Difference in potential WLE**  **(in years)** | **Components** | | | | | | |  |
| --- | --- | --- | --- | --- | --- | --- | --- | --- | --- | --- | --- |
|  |  |  |  | **Employment** | | **Working**  **hours** | **Unemployment** | | | **Unfulfilled/Overful-filled working time desires*** | |
| Men | West | high | - | Reference: 41.6 years | | | | | | |  |
|  |  | medium | -0,8 | -1,3 | 0,0 | | | 0,4 | - | |  |
|  |  | low | -3,2 | -4,8 | 0,0 | | | 1,6 | - | |  |
|  | East | high | -0,9 | -2,6 | 0,0 | | | 1,7 | - | |  |
|  |  | medium | -0,3 | -2,8 | 0,1 | | | 2,4 | - | |  |
|  |  | low | -3,4 | -7,2 | 0,0 | | | 3,8 | - | |  |
| Women | West | high | - | Reference: 29.2 years | | | | | | |  |
|  |  | medium | -4.0 | -2,6 | -1,2 | | | -0,2 | - | |  |
|  |  | low | -8.5 | -7,9 | -0,7 | | | 0,1 | - | |  |
|  | East | high | +7.1 | 2,5 | 3,7 | | | 0,9 | - | |  |
|  |  | medium | +5.3 | -0,1 | 2,4 | | | 2,9 | - | |  |
|  |  | low | -0.1 | -4,7 | 1,5 | | | 3,2 | - | |  |

* Data on Unfulfilled/Overfulfilled working time desires is not available for the year 1991.

Source: RDC of the Federal Statistical Office and the Statistical Offices of the Federal States of Germany, Microcensus 1991, authors’ calculations.

**Table A5b: Results of the decomposition analysis of the differences in potential working life expectancy (WLE) by gender, region (eastern/western Germany), and level of education, 2001**

| **Gender** | **Region** | **Education level** | **Difference in potential WLE**  **(in years)** | **Components** | | | | | | |  |
| --- | --- | --- | --- | --- | --- | --- | --- | --- | --- | --- | --- |
|  |  |  |  | **Employment** | | **Working**  **hours** | **Unemployment** | | | **Unfulfilled/Overful-filled working time desires*** | |
| Men | West | high | - | Reference: 42.0 years | | | | | | |  |
|  |  | medium | -2,8 | -3,4 | -0,4 | | | 1,0 | - | |  |
|  |  | low | -4,7 | -8,4 | -0,3 | | | 3,9 | - | |  |
|  | East | high | -2,0 | -3,2 | -0,6 | | | 1,8 | - | |  |
|  |  | medium | -3,8 | -9,6 | -0,3 | | | 6,0 | - | |  |
|  |  | low | -7,8 | -16,9 | -0,6 | | | 9,8 | - | |  |
| Women | West | high | - | Reference: 31.1 years | | | | | | |  |
|  |  | medium | -5,7 | -3,3 | -2,6 | | | 0,2 | - | |  |
|  |  | low | -12,2 | -10,3 | -2,8 | | | 0,8 | - | |  |
|  | East | high | 3,7 | -0,7 | 2,7 | | | 1,8 | - | |  |
|  |  | medium | 0,1 | -5,8 | 0,5 | | | 5,3 | - | |  |
|  |  | low | -7,1 | -13,8 | -0,4 | | | 7,2 | - | |  |

* Data on Unfulfilled/Overfulfilled working time desires is not available for the year 2001.

Source: RDC of the Federal Statistical Office and the Statistical Offices of the Federal States of Germany, Microcensus 2001, authors’ calculations.

**Table A5c: Results of the decomposition analysis of the differences in potential working life expectancy (WLE) by gender, region (eastern/western Germany), and level of education, 2011**

| **Gender** | **Region** | **Education level** | **Difference in potential WLE**  **(in years)** | **Components** | | | | | | |  |
| --- | --- | --- | --- | --- | --- | --- | --- | --- | --- | --- | --- |
|  |  |  |  | **Employment** | | **Working**  **hours** | **Unemployment** | | | **Unfulfilled/Overful-filled working time desires** | |
| Men | West | high | - | Reference: 42.8 years | | | | | | |  |
|  |  | medium | -2,6 | -3,9 | -0,3 | | | 1,1 | 0,5 | |  |
|  |  | low | -5,7 | -10,2 | -0,8 | | | 4,0 | 1,3 | |  |
|  | East | high | -2,1 | -2,4 | -0,6 | | | 0,5 | 0,4 | |  |
|  |  | medium | -3,7 | -7,6 | -0,5 | | | 3,4 | 1,0 | |  |
|  |  | low | -11,9 | -18,9 | -1,8 | | | 7,1 | 1,7 | |  |
| Women | West | high | - | Reference: 33.5 years | | | | | | |  |
|  |  | medium | -6,2 | -3,4 | -3,6 | | | 0,4 | 0,5 | |  |
|  |  | low | -13,6 | -11,8 | -4,0 | | | 1,4 | 0,8 | |  |
|  | East | high | 2,9 | -0,1 | 2,2 | | | 0,4 | 0,4 | |  |
|  |  | medium | -1,9 | -5,1 | -0,8 | | | 2,3 | 1,7 | |  |
|  |  | low | -11,6 | -15,5 | -1,7 | | | 4,4 | 1,3 | |  |

Source: RDC of the Federal Statistical Office and the Statistical Offices of the Federal States of Germany, Microcensus 2011, authors’ calculations.
